# Modeling and Experimental Investigation of Ultrasonic Vibration-Assisted Drilling Force for Titanium Alloy

**DOI:** 10.3390/ma18194460

**Published:** 2025-09-24

**Authors:** Chuanmiao Zhai, Xubo Li, Cunqiang Zang, Shihao Zhang, Bian Guo, Canjun Wang, Xiaolong Gao, Yuewen Su, Mengmeng Liu

**Affiliations:** 1 Shaanxi Key Laboratory of Advanced Manufacturing and Evaluation of Robot Key Components, College of Mechanical Engineering, Baoji University of Arts and Sciences, Baoji 721016, China; 18237358753@163.com (C.Z.);; 2 Zhejiang Sankai Mechanical and Electrical Co., Ltd., Taizhou 317511, China; 3 School of Mechanical and Precision Instrument Engineering, Xi’an University of Technology, Xi’an 710048, China

**Keywords:** ultrasonic vibration-assisted drilling, cutting force model, dynamic cutting thickness, finite element simulation, twist drill

## Abstract

To overcome the issues of excessive cutting force, poor chip segmentation, and premature tool wear during the drilling of Ti-6Al-4V titanium alloy. This study established the cutting edge motion trajectory function and instantaneous dynamic cutting thickness equation for ultrasonic vibration-assisted drilling through kinematic analysis. Based on this, an analytical model of drilling force was formulated, integrating tool geometry, cutting radius scale effects, dynamic chip thickness, and drilling depth. In parallel, a finite element model was constructed to achieve visual simulation analysis of chip deformation and cutting force. Finally, the accuracy of the model was verified through experiments, with a comprehensive analysis performed on how cutting parameters affect thrust force. The findings indicate that the average absolute prediction errors of thrust force and torque between the analytical model and finite element simulations were 7.87% and 6.26%, respectively, confirming the model’s capability to accurately capture instantaneous force and torque variations. Compared to traditional drilling methods, the application of ultrasonic vibration assistance resulted in reductions of 40.8% in thrust force and 41.7% in torque. The drilling force exhibited nonlinear growth as the spindle speed and feed rate were elevated, while it declined with greater vibration frequency and amplitude as drilling depth increased. Furthermore, the combined effect of optimized vibration parameters enhanced chip fragmentation, producing short discontinuous chips and effectively preventing entanglement. Overall, this research provides a theoretical and practical foundation for optimizing ultrasonic vibration-assisted drilling and improving precision hole making in titanium alloys.

## 1. Introduction

Titanium alloys are known as “all-round metals” and “space metals” owing to their exceptional combination of low density, superior strength, excellent corrosion resistance, high-temperature tolerance, and excellent low-temperature performance, and are extensively employed in high-tech industries including medical devices, aerospace, and marine engineering [1,2]. Nevertheless, their poor thermal conductivity, high plasticity, and low elastic modulus characteristics of the material during machining make it a typical hard-to-cut material, and during semi-closed cutting operations such as drilling and tapping, there will be difficulties in chip breakage, serious tool adhesion wear, and intermittent edge accumulation, which directly affects the machining accuracy and drilling efficiency [3,4]. In drilling and machining, it often faces problems such as chip winding around tools, poor surface quality, and rapid tool wear. To solve these challenges, ultrasonic vibration-assisted drilling (UVAD) technology has emerged, which usually refers to the use of ultrasonic waves with vibration frequencies higher than 15 kHz to assist drilling [5,6]. Compared to conventional drilling (CD), ultrasonic vibration-assisted drilling has the advantages of reducing cutting force and friction, improving hole surface quality, promoting chip breaking and evacuation, and improving machining efficiency [7,8,9], providing an effective method for improving both quality and efficiency in the field of machining, especially when machining difficult-to-machine materials.

Regarding the mechanism of ultrasonic vibration-assisted drilling, Pu et al. [10] studied titanium alloy ultrasonic vibration-assisted drilling, observed chip morphology through high-speed cameras, analyzed tool temperature changes through infrared temperature measurement, and revealed the influence of ultrasonic vibration-assisted drilling on the reduction in cutting force and temperature increase. Huang et al. [11] investigated the wear characteristics of twist drills in ultrasonic vibration-assisted drilling of carbon fiber reinforced plastics (CFRP). They demonstrated that ultrasonic assistance effectively reduces tool wear and extends tool life compared with conventional drilling. Chen et al. [12], through theoretical modeling and experimental research, established a theoretical domain of geometric chip breaking conditions, and the advantages of ultrasonic vibration-assisted drilling technology in terms of chip breaking effects, surface roughness, and tool wear have been verified through experiments. Lu et al. [13] established a model for predicting cutting forces for longitudinal torsion ultrasonic drilling (LTUM) and established the tool trajectory equation. Through kinematic analysis, they studied the dynamic cutting characteristics, and compared experiments showed that the cutting force of longitudinal torsion compound ultrasonic vibration-assisted drilling was markedly higher than in conventional drilling operations. Wei et al. [14] combined Deform-3D simulations with experimental validation to investigate the micro-drilling performance of titanium alloys and aluminum alloys. They demonstrated that ultrasonic assistance can markedly suppress interlayer damage, while simultaneously lowering thrust force and drilling temperature.

Despite these advantages, the mechanical behavior of ultrasonic vibration-assisted drilling remains complex, as its dynamic cutting forces are strongly influenced by machining parameters, material characteristics, and the kinematics of ultrasonic motion [15,16]. Existing approaches for modeling cutting forces generally fall into four categories: geometric analysis, empirical parameter-based models, finite element simulations, and analytical methods. Among them, the development of a precise analytical model is regarded as essential for optimizing process conditions, predicting cutting forces, minimizing tool wear, and improving overall machining quality [17]. Consequently, researchers have proposed various theoretical models and numerical simulation techniques.

In the field of cutting force modeling, Ji et al. [18] established both a mathematical prediction framework and a finite element simulation model to investigate thrust force during ultrasonic vibration-assisted drilling of SiCp/AL6063 composites. Their experimental findings verified the effectiveness of ultrasonic assistance in lowering cutting forces and enhancing chip morphology. Nonetheless, the proposed drilling force model did not incorporate the dynamic variations in cutting layer thickness or tooth angle. Wan et al. [19] introduced a consolidated real-time machining force formulation and, by applying geometric transformation and bevel-cutting theory, developed a general prediction approach applicable to flat-end milling cutters. They further calibrated critical parameters, including shear stress and friction angle, using a limited number of milling experiments. The model has made significant contributions in terms of generality, but neglecting the time-varying factors in the processing may affect its long-term prediction stability. Yang et al. [20] constructed an analytical model for thrust force for drilling SiCp/Al composites microhole considering vibration acceleration and undeformed chip thickness, but did not consider the dynamic change in the blade angle along the cutting radius, and could not predict the change in instantaneous drilling force. Yang et al. [21] constructed a thrust dynamic model under low-frequency vibration drilling of titanium alloys. They further introduced the concept of a critical chip-breaking amplitude and optimized the corresponding drilling parameters. However, the thermo-mechanical coupling effect of titanium alloy in the vibration drilling process has not been adequately considered. Li et al. [22] implemented low-frequency axial vibration-assisted BTA deep-hole machining using an eccentric cam mechanism. Through reverse analysis, they determined the distribution of blade cutting force with respect to cutting radius and dynamic cutting thickness. However, the model does not fully consider the impact of the coupling effects of vibration drilling process parameters on the axial force during drilling.

Yuan et al. [23] established the CFRP-T700 ultrasonic vibration-assisted drilling cutting force model founded on the principles of brittle fracture mechanics, introduced the indentation fracture theory and single-factor experimental calibration parameter *K* value. However, the model lacks sufficient analysis of the dynamic fracture process of fibers and the instantaneous force changes caused by vibrations. Matsumura et al. [24] established a predictive modeling framework for drilling forces for CFRP/titanium alloy stacks by combining orthogonal and bevel cutting theories, explicitly considering the influence of interlayer transitions. However, insufficient consideration of the influence of interlayer interactions on changes in cutting force restricts the predictive accuracy of the model in multi-interface layered processing. Elhachimi et al. [25] proposed a mechanical model for high-speed drilling that predicts thrust force and torque through angular cutting and orthogonal cutting theories, without fully considering the effect of strain rate under high-speed conditions. Wu et al. [26] developed a thrust force model for Ti-6Al-4V drilling that incorporates feed effects, refined the equivalent treatments of bevel and orthogonal cutting, and introduced a partitioned model distinguishing the main and chisel cutting edges. However, there is insufficient consideration of the dynamic effects of cutting force caused by tool wear. Li et al. [27] established a model for dynamic cutting forces in ultrasonic vibration-assisted drilling using micro-element analysis and dynamic fiber orientation evaluation, demonstrating that ultrasonic vibration reduces peak cutting forces and minimizes delamination. However, there is insufficient consideration of the coupling effects between ultrasonic vibration parameters and fiber orientation.

Existing modeling of cutting forces mostly does not consider that the cutting force along the drilling radius is not a fixed value. In the process of ultrasonic vibration drilling, the instantaneous effective rake angle, cutting layer thickness, and shear angle of the cutting teeth are dynamically changing, and the cutting force coefficient is also not constant. There is a lack of prediction and research on the instantaneous cutting force during ultrasonic vibration drilling, making it difficult to accurately describe the synergistic effects of various process parameters such as vibration frequency, amplitude, spindle speed, and feed rate on the cutting force. Therefore, this paper takes titanium alloy ultrasonic vibration-assisted drilling as the research object, and establishes a drilling force analytical model considering tool geometric parameters, cutting radius scale effect, dynamic cutting thickness, and drilling depth based on the kinematic analysis of ultrasonic vibration-assisted drilling and tool geometric parameters.

The structure of this paper is composed of the following sections: a literature review in the introduction section; an analysis of cutting mechanisms in the kinematics analysis section; an analysis of drilling force models in the models section; a visualization analysis of the drilling process achieved through finite element simulation in the simulation section; parameter identification and model verification in the experimental section; a detailed discussion of the theory-simulation-experiment of the model in the analysis and discussion section; and a conclusion section that comprehensively summarizes the research results and limitations of this paper.

## 2. Kinematics Analysis

**Figure 1:**
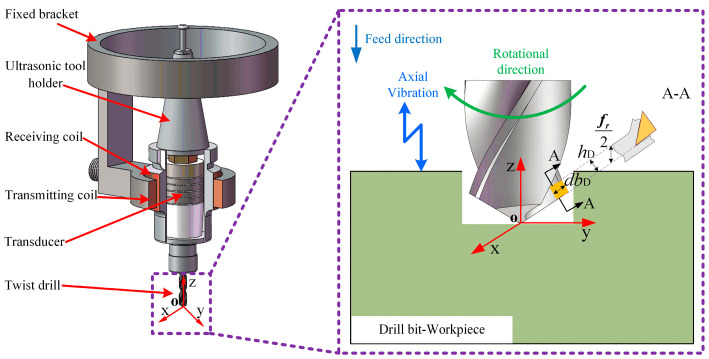
Ultrasonic vibration-assisted drilling principle and ultrasonic tool holder.

Ultrasonic vibration-assisted drilling evolves from conventional drilling by equipping the cutting tool with a high-frequency vibration system, which imparts an axial oscillation during machining and produces a compound cutting motion. The vibration unit generally comprises an ultrasonic generator, a transducer, and an ultrasonic tool holder, enabling axial oscillations typically above 15 kHz. Under these conditions, the tool operates the feed motion while superimposing low-amplitude, high-frequency axial vibrations. This combined motion periodically alters the dynamics at the tool-workpiece interface, thereby modifying the cutting mechanism. The schematic principle of UVAD is illustrated in Figure 1.

In conventional drilling, there is a continuous and stable contact between the drill bit and the workpiece, which often leads to large cutting forces and heat buildup, which negatively affects the machining quality. In ultrasonic vibration-assisted drilling, due to the existence of vibration, the contact between the drill bit and the workpiece is no longer continuous, but presents a periodic intermittent contact mode of contact-disconnection-contact. Under the influence of ultrasonic vibrations, the cutter and the workpiece experience intermittent contact and separation, effectively reducing both friction and cutting resistance during drilling. As a result, the process not only decreases cutting forces and suppresses heat generation but also significantly enhances the surface integrity of the hole wall. Furthermore, chip accumulation within the hole is alleviated, leading to smoother chip evacuation and improved overall machining efficiency.

**Figure 2:**
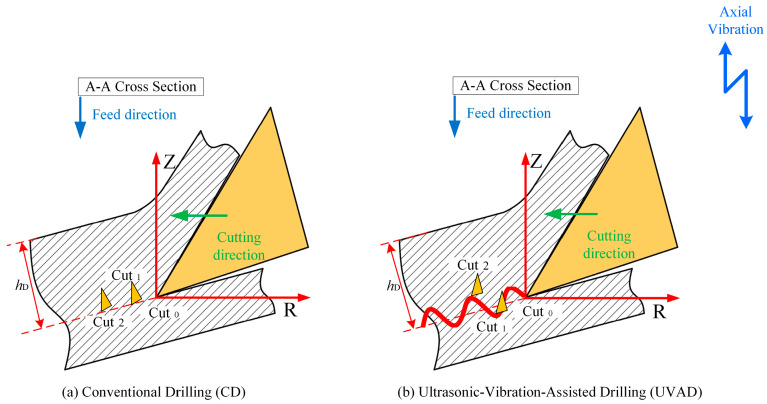
Drilling process layout along the A-A section of the main cutting edge.

Figure 2 shows a simplified diagram of the drilling process along the A-A section of the main cutting edge, and the high-frequency vibration is output to the tool through an ultrasonic vibration tool holder, so that the movement mode of the tool is composed of axial feed motion, axial sinusoidal vibration, and circumferential rotational motion, so the total axial positional offset of the tool with respect to the workpiece is

(1) $$Z\left(t\right)=nf_{r}t/60+A\sin\left(2\pi ft\right)$$

where *n* is the spindle speed (r/min); *f*_r_ is the feed volume (mm/r); *t* is the drilling time (s); *A* is the amplitude (μm); *f* is the vibration frequency (Hz).

When the drill bit makes a rotational feed motion relative to the workpiece, the relationship between the circumferential angular displacement *θ* and time *t* can be expressed as

(2) $$\theta \left(t\right)=2\pi t\left(\frac{n}{60}\right)=n\pi t/30$$

Assuming that the P distance of any point on the main cutting edge of the drill bit is *r*, and the unit is (mm), then the equations of motion at any time on the main cutting edge during the drilling process are

(3) $$\left\{\begin{array}{l} X=r\cos\left(2\pi nt\right)\\ Y=r\cos\left(2\pi nt\right)\\ Z=nf_{r}t/60+A\sin\left(2\pi nt\right) \end{array}\right.$$

**Figure 3:**
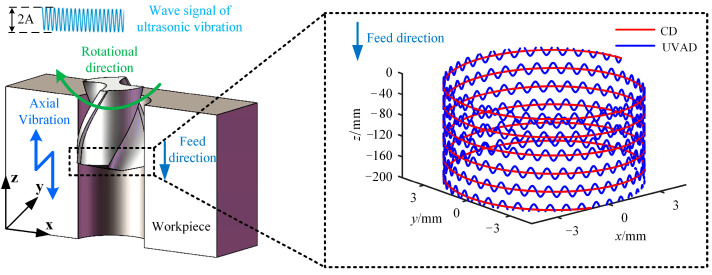
Movement trajectory of the main cutting edge of UVAD.

According to the simulation conditions, the following parameters are selected for the cutting edge trajectory analysis: *n* = 1500 r/min, *f*_r_ = 0.05 mm/r, *A* = 6 μm, *f* = 20,010 Hz, *r* = 3 mm. Substituting these parameters into Equation (3), the motion trajectory of any point P on the main cutting edge of the drill bit is shown in Figure 3.

As illustrated in Figure 3, the trajectory of ultrasonic vibration-assisted drilling generally follows a downward path along the tool axis, yet it deviates from a smooth curve and instead exhibits a sinusoidal form with periodic oscillations. Relative to the conventional drilling path in Figure 3, this trajectory displays a displacement offset between two successive cutting paths. Moreover, in contrast to traditional drilling, the axial spacing of the primary cutting edges in ultrasonic vibration-assisted drilling varies dynamically, leading to periodic fluctuations in instantaneous chip thickness with the angular rotation of the drill bit.

Since the configuration of a twist drill consists of two symmetrically arranged main cutting edges and a central chisel edge, a full drilling cycle corresponds to a 180° rotation of the tool, which results in a circumferential angular difference of π across equivalent positions on the pair of tool blades. According to Equations (1) and (2), the motion of these two points can thus be expressed mathematically as follows:

(4) $$\left\{\begin{array}{l} Z_{m}\left(\theta \right)=\frac{f_{r}\theta }{2\pi }+A\sin\left(\frac{60f\theta }{n}\right)\\ Z_{n}\left(\theta \right)=\frac{f_{r}\left(\theta +\pi \right)}{2\pi }+A\sin\left(\frac{60f\left(\theta +\pi \right)}{n}\right) \end{array}\right.$$

The axial offset between the primary cutters is the dynamic cutting thickness of *h* during ultrasonic vibration-assisted drilling, which can be expressed as

(5) $$\begin{array}{l} h\left(\theta \right)=Z_{m}\left(\theta \right)-Z_{n}\left(\theta \right)\\ \;\;\;\;\;\;\;\;=\frac{f_{r}}{2}+2A\cos\left(\frac{60f\theta }{n}+\frac{30f\pi }{n}\right)\sin\left(\frac{30f\pi }{n}\right) \end{array}$$

When ultrasonic vibration-assisted drilling, the minimum axial dynamic cutting thickness *h*_min_ is

(6) $$h_{\min}=\frac{f_{r}}{2}-2A\left|\sin\left(\frac{30\pi f}{n}\right)\right|$$

**Figure 4:**
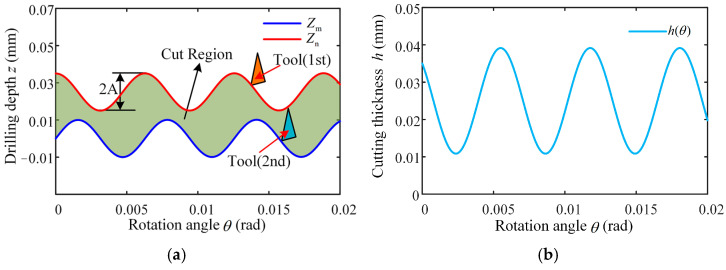
Cutting edge trajectory and dynamic cutting thickness changes in the tool: (**a**) two-dimensional cutting trajectory of UVAD; (**b**) dynamic theoretical cutting thickness.

Equation (5) indicates that when adjacent cutting paths in ultrasonic vibration-assisted drilling exhibit a phase shift, the chip thickness ceases to remain constant and instead varies periodically, which is the dynamic cutting characteristics of vibration drilling. According to Equation (6), we can reasonably select the drilling parameters so that the minimum cutting thickness can be reduced to zero or even negative, so as to promote the fracture of chips. Figure 4 illustrates the cutting path of the tool edge in ultrasonic vibration-assisted drilling, along with the corresponding variations in dynamic chip thickness, and the cutting thickness fluctuates with the change in the machining process. Consequently, the chip thickness varies periodically, following a sinusoidal pattern centered around half the feed. This dynamic change allows the cutting thickness to change continuously, which in turn helps to improve chip breaking.

## 3. Model

**Figure 5:**
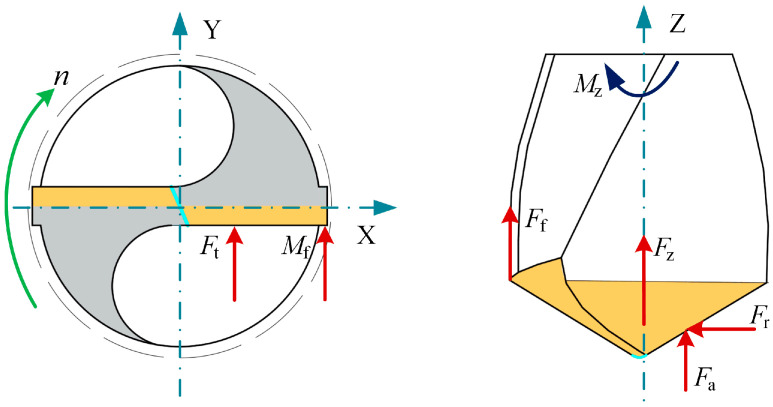
The force acting on the blade of the tool.

Figure 5 presents the composition of drilling forces for a twist drill. The thrust force exerted on the drill bit can be divided into three force components acting along the main cutting edge and the thrust generated by the chisel edge. Since a standard twist drill is geometrically symmetric, the analysis typically focuses on only one of the two primary cutting edges. The associated thrust force and torque of the drill can therefore be expressed as follows:

(7) $$F_{Z}=F_{Z1}+F_{Z2}+F_{f}$$

(8) $$M_{Z}=M_{Z1}+M_{Z2}+M_{f}$$

where *F*_Z1_ represents the axial thrust applied on the main cutting edge, while *F*_Z2_ corresponds to the thrust produced by the chisel edge. The term *F*_f_ denotes the thrust force resulting from the rubbing of the secondary cutting edge against the borehole wall. *M*_Z1_ refers to the torque acting on the main cutting edge, and *M*_Z2_ describes the torque generated by the chisel edge. *M*_f_ signifies the torque caused by the frictional interaction between the secondary cutting edge and the borehole surface.

**Figure 6:**
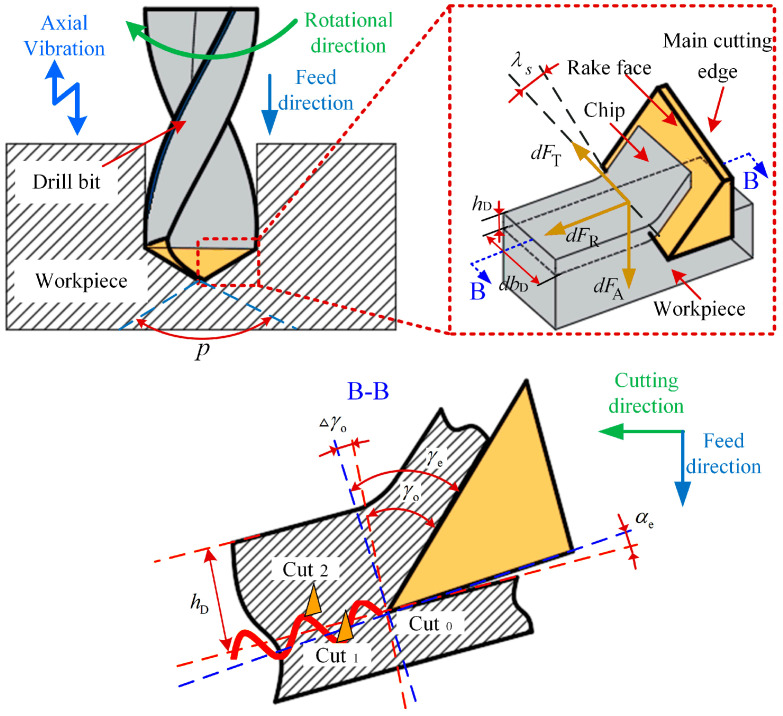
Micro-element cutting force model of the main cutting edge of the twist drill tool.

Based on the geometric interaction between the drill’s primary cutting zone and the machined material, the edge is discretized into a series of micro-cutting elements. Each element is treated as an independent oblique cutting unit, and the overall thrust force and torque are determined through the integration of individual force components along the cutting radius. Owing to the large geometric angles of twist drills, the main cutting edge incorporates a cutting angle in the central region where the cutting speed approaches zero. The corresponding micro-element force model is depicted in Figure 6.

The load acting on each cutting micro-element can be expressed as the product of the relevant coefficient for cutting force, together with the differential area element. Accordingly, the cutting force generated on the tooth micro-element of the main cutting edge is formulated as

(9) $$\left\{\begin{array}{l} dF_{T}=K_{T}\cdot h_{D}\cdot db_{D}\\ dF_{R}=K_{R}\cdot h_{D}\cdot db_{D}\\ dF_{A}=K_{A}\cdot h_{D}\cdot db_{D} \end{array}\right.$$

where *h*_D_ is the thickness of the cutting layer; *db*_D_ is the cutting width; *K*_T_, *K*_R_ and *K*_A_ are the tangential, radial and axial cutting force coefficients, respectively. *dF*_T_, *dF*_R_, and *dF*_A_ are the tangential, radial, and thrust forces of the cutting edge microelements, respectively.

According to the bevel cutting theory, the modified cutting force coefficient is derived as [28]

(10) $$\left\{\begin{array}{l} K_{T}=\frac{\tau_{s}}{\sin\phi_{n}}\frac{\cos\left(\beta_{n}-\gamma_{n}\right)+\tan\lambda_{s}\tan\eta_{c}\sin\beta_{n}}{\sqrt{\cos^{2}\left(\phi_{n}+\beta_{n}-\gamma_{n}\right)+\tan^{2}\eta_{c}\sin^{2}\beta_{n}}}\\ K_{R}=\frac{\tau_{s}}{\sin\phi_{n}}\frac{\cos\left(\beta_{n}-\gamma_{n}\right)\tan\lambda_{s}-\tan\eta_{c}\sin\beta_{n}}{\sqrt{\cos^{2}\left(\phi_{n}+\beta_{n}-\gamma_{n}\right)+\tan^{2}\eta_{c}\sin^{2}\beta_{n}}}\\ K_{A}=\frac{\tau_{s}}{\sin\phi_{n}\cos\lambda_{s}}\frac{\sin\left(\beta_{n}-\gamma_{n}\right)}{\sqrt{\cos^{2}\left(\phi_{n}+\beta_{n}-\gamma_{n}\right)+\tan^{2}\eta_{c}\sin^{2}\beta_{n}}} \end{array}\right.$$

where *τ*_s_ is the shear stress; *ϕ*_n_ is the normal shear angle; *β*_n_ is the normal friction angle; *λ*_s_ is the angle of inclination of the blade; *η*_c_ is the angle of the flow chip; *γ*_n_ refers to the actual angle of attack under standard cutting conditions; where *γ*_n_ = arctan(tan*γ*_e_ · cos*η*_c_). Established by Stabler, the principle dictates that the chip flow angle is defined by the blade’s inclination, *η*_c_ = *λ*_s_.

**Figure 7:**
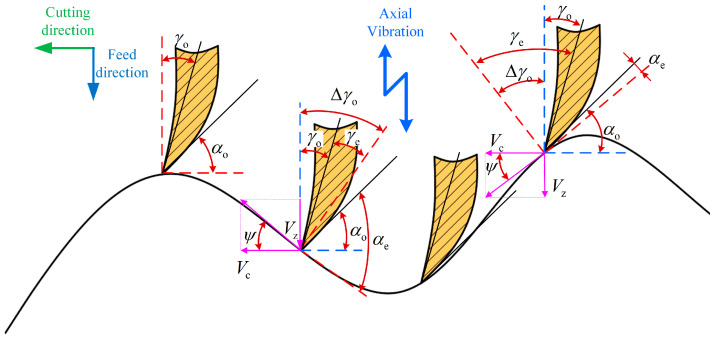
The instantaneous cutting angle of UVAD.

In ultrasonic vibration-assisted drilling, both the cutting speed and the angle of the tool’s main cutting edge vary continuously. A schematic illustrating the dynamic cutting angle of the micro-element during the cutting process is presented in Figure 7. The actual cutting geometry in operation can be represented as

(11) $$\gamma_{e}=\gamma_{o}+△\gamma_{o}=\gamma_{o}+\psi$$

The real-time cutting angle can be formulated as

(12) $$\psi =\arctan\frac{V_{Z}}{V_{C}}$$

Among them, the axial dynamic cutting speed is

(13) $$V_{Z}=nf_{r}/60+2\pi Af\cos\left(2\pi nt\right)$$

where the unit of axial dynamic cutting speed is mm/s.

The tangential speed that the main cutting edge undergoes is

(14) $$V_{C}=2\pi rn/60$$

where the unit of tangential speed is mm/s.

By combining Equations (2), (13) and (14), we can obtain

(15) $$\psi =\arctan\left(\frac{\frac{60Af}{n}\cdot \cos\left(\frac{60f}{n}\cdot \theta \right)}{r}+\frac{f_{r}}{2\pi r}\right)$$

The dynamic cutting layer thickness is

(16) $$h_{D}=h\left(\theta \right)\cdot \cos K_{r}=\left(\frac{f_{r}}{2}+2A\cos\left(\frac{60f\theta }{n}+\frac{30f\pi }{n}\right)\sin\left(\frac{30f\pi }{n}\right)\right)\cdot \cos K_{r}$$

On the cutting microelement *dr* distributed along the drilling radius, the corresponding cutting width is

(17) $$db_{D}=\frac{dr}{\cos K_{r}}$$

where *f*_r_ is the feed per revolution during the drilling process; *K*_r_ is the cutting edge angle, *K*_r_ = (π − *p*)/2, and *p* is the point angle of the twist drill.

During drilling operations enhanced by ultrasonic vibration, the thickness, shear angle, and friction angle of the cutting layer on the cutting tooth along the cutting radius vary dynamically. As reported in Ref. [22], the angle of friction in the normal direction and the corresponding shear angle can be expressed as functions of the dynamic cutting thickness and the tool’s cutting radius, given by Equations (18) and (19), respectively. An increase in the normal friction angle is observed when the real-time cutting thickness and radius are reduced. Conversely, the growth in undeformed chip thickness and tool engagement radius leads to an expansion of the orthogonal shear angle.

(18) $$\beta_{n}(h_{D},r)=2.21{h_{D}}^{2}+0.001553r^{2}+0.03507h_{D}\cdot r-1.722h_{D}-0.03287r+0.618$$

(19) $$\phi_{n}(h_{D},r)=-2.21{h_{D}}^{2}-0.002316r^{2}-0.03475h_{D}\cdot r+1.718h_{D}+0.0395r+0.1668$$

By substituting Equations (10), (16) and (17) into Equation (9), the tangential, radial, and thrust forces acting on a microelement of the cutting edge can be determined. Through a coordinate transformation of the triaxial forces within the cutting edge coordinate system and by rotating the main cutting edge declination angle *K*_r_ in the direction of the *dF*_T_ force, the element-specific reference frame for the tool segment is transformed into the tool coordinate system. This transformation can be expressed as

(20) $${\left[\begin{array}{ccc} dF_{t} & dF_{r} & dF_{a} \end{array}\right]}^{T}=T\left(K_{r}\right){\left[\begin{array}{ccc} dF_{T} & dF_{R} & dF_{A} \end{array}\right]}^{T}$$

(21) $$T\left(K_{r}\right)=\left[\begin{array}{ccc} 1 & 0 & 0\\ 0 & \cos K_{r} & \sin K_{r}\\ 0 & -\sin K_{r} & \cos K_{r} \end{array}\right]$$

Consequently, the main cutting edge produces both the thrust force and the torque during the cutting of the workpiece are

(22) $$F_{Z1}=2\int_{r_{1}}^{R}dF_{a}$$

(23) $$M_{Z1}=2\int_{r_{1}}^{R}rdF_{t}$$

where *R* is the radius of the twist drill tool, and *r*_1_ is the radius of the edge of the chisel edge.

**Figure 8:**
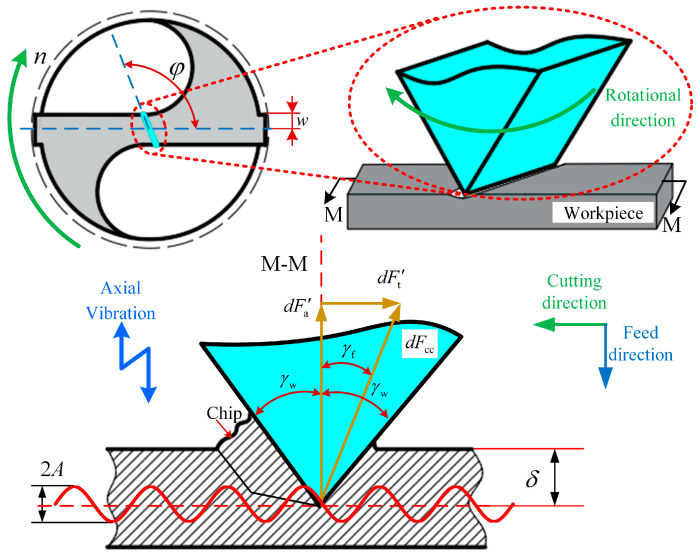
Microelement extrusion force model of the chisel edge of the twist drill tool.

Because the cutting speed at the chisel edge of a twist drill is relatively low, material removal in this region primarily occurs through extrusion. The chisel edge is discretized into equal microelements along the drilling radius [29]. The extrusion model of these microelements on the twist drill cross blade is illustrated in Figure 8.

The microelement extrusion force on the chisel edge of a twist drill is expressed as

(24) $$dF_{\mathrm{cc}}=E_{3}^{*}\delta \tan\gamma_{w}$$

where *E*_3_* is the equivalent Young’s modulus, *E*_3_* = *E*_3_/(1 − *μ*^2^), where *E*_3_ is the Young’s modulus of the titanium alloy Ti-6Al-4V, and *μ* is the Poisson’s ratio value for the titanium alloy. *δ* is the extrusion depth of the chisel edge, *δ* = *δ*_0_ + *h*_D_, where *δ*_0_ = *k*_cc_*w*, *w* is the semi-width of the chisel edge, and *k*_cc_ is the chisel edge coefficient, according to Ref. [21], *k*_cc_ is 0.001. *γ*_w_ is the cutting geometry at the chisel edge’s engagement zone, *γ*_w_ = arctan(tan*p* ⋅ sin*φ*), where *φ* is the rake angle of the chisel edge.

Since the chisel edge rotates during drilling, the cutting speed acts perpendicular to the chisel edge, and the micro element extrusion force *dF*_cc_ is deflected around the feed angle *γ*_f_, where *γ*_f_ = arctan(*h*_D_/*πR*). *dF*_cc_ can be decomposed into its thrust force component $dF_{a}^{\prime }$ and tangential force component $dF_{t}^{\prime }$ are

(25) $$dF_{a}^{\prime }=dF_{\mathrm{cc}}\cos\gamma_{f}$$

(26) $$dF_{t}^{\prime }=dF_{\mathrm{cc}}\sin\gamma_{f}$$

The thrust force and torque generated from the chisel edge are defined by the following expressions:

(27) $$F_{Z2}=2\int_{0}^{r_{1}}dF_{a}^{\prime }dr=2\int_{0}^{r_{1}}dF_{\mathrm{cc}}\cos\gamma_{f}dr$$

(28) $$M_{Z2}=2\int_{0}^{r_{1}}dF_{t}^{\prime }rdr=2\int_{0}^{r_{1}}dF_{\mathrm{cc}}\sin\gamma_{f}rdr$$

In the drilling process, the minor cutting edge of the twist drill produces friction *F*_f_ with the inner wall of the drilling hole, the direction of the force is the opposite direction of the cutting speed direction, and the magnitude of the force is related to the drilling depth. Assuming that the friction between the minor cutting edge and the hole wall has the same force amplification factor (FAF) for drilling torque and thrust force, it is *K*_L_. Therefore, as drilling progresses to greater depth *L*, the incremental function of the effects of the auxiliary cutting edge on force and torque output is

(29) $$\xi \left(L\right)=1+K_{L}\cdot \left(L-L_{0}\right)$$

where *K*_L_ is the FAF, *L* is the drilling depth (mm), and *L*_0_ is the vertical distance between the transverse edge and the minor cutting edge, which is expressed as

(30) $$L_{0}=\frac{D}{2\tan\frac{p}{2}}$$

where *D* is the diameter of the twist drill.

Hence, the resultant thrust force and torque during ultrasonic vibration-assisted drilling are

(31) $$\begin{array}{l} F_{Z}=\left({F_{Z}}_{1}+F_{Z2}\right)\cdot \xi \left(L\right)\\ \;\;\;\;\;=\left(2\int_{r_{1}}^{R}dF_{a}+2\int_{0}^{r_{1}}dF_{a}^{\prime }dr\right)\cdot \left(1+K_{L}\cdot \left(L-\frac{D}{2\tan\frac{p}{2}}\right)\right) \end{array}$$

(32) $$\begin{array}{l} M_{Z}=\left({M_{Z}}_{1}+M_{Z2}\right)\cdot \xi \left(L\right)\\ \;\;\;\;\;\;=\left(2\int_{r_{1}}^{R}rdF_{t}+2\int_{0}^{r_{1}}dF_{t}^{\prime }rdr\right)\cdot \left(1+K_{L}\cdot \left(L-\frac{D}{2\tan\frac{p}{2}}\right)\right) \end{array}$$

## 4. Simulation

**Figure 9:**
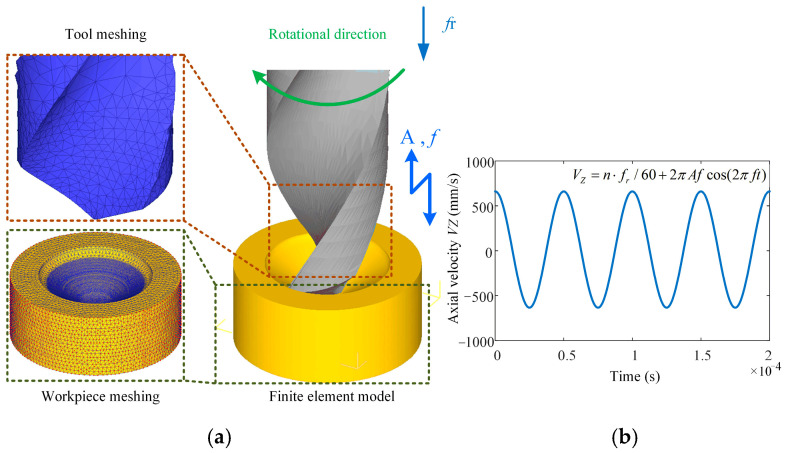
Meshing model of tools and workpieces: (**a**) FEM simulation model; (**b**) instantaneous axial velocity.

As a powerful tool for nonlinear analysis, the finite element method can solve many complex problems that cannot be directly conducted in the laboratory due to conditions. A simulation model developed using finite element principles was established in this research to simulate ultrasonic vibration-assisted drilling with DEFORM-3D v11.0 software. Due to the complexity of the tool’s geometric structure, the mesh division size is relatively small and numerous. The workpiece modeling is simplified to a concave structure, omitting the simulation of the drilling process, allowing the main cutting edge to fully participate in cutting from the start of the simulation. After many experiments and theoretical analysis, the results show that when the diameter of the workpiece is slightly larger than 20% of the drill bit diameter, the simulation accuracy can still be effectively guaranteed [30,31]. Therefore, in this study, the workpiece was set as a cylindrical concave blank structure featuring a 9 mm diameter and a 4 mm thickness. The established tool and workpiece simulation model is shown in Figure 9a. The tool geometry parameters are shown in Table 1.

In conducting the simulation of ultrasonic vibration-assisted drilling, it is necessary to superimpose a sinusoidal vibration onto the axial feed motion. The ultrasonic vibration feed is calculated according to Equation (13). The velocity function of simple harmonic vibration is approximated to the shape of the sine wave so that the ultrasonic vibration effect during the actual drilling process can be more accurately simulated. Figure 9b presents an image of this velocity function, visualizing the fluctuation of the feed over time.

The constitutive model serves as a mathematical framework for characterizing the deformation and mechanical behavior of materials by defining the stress–strain relationship within the cutting zone. This enables the prediction of stress distribution and strain accumulation, thereby facilitating analysis of the mechanical response during material removal. Selecting and formulating an appropriate constitutive model is essential for enhancing the precision and reliability of drilling simulations. In this study, the workpiece material is Ti-6Al-4V, whose mechanical properties are represented using the Johnson–Cook (J–C) constitutive model. The reference values of the J–C were obtained by reviewing a large number of literature, filtering parameters to select those with high citations and consistent with the methods used in the simulation part of this Ref. [32]. The mathematical representation of the model is given as

(33) $$\sigma =\left(A+B\varepsilon^{u}\right)\left(1+C\ln\frac{\dot{\varepsilon }}{{\dot{\varepsilon }}_{0}}\right)\left[1-{\left(\frac{T-T_{0}}{T_{\mathrm{melt}}-T_{0}}\right)}^{m}\right]$$

where *A* denotes the yield stress under static loading conditions; *B* denotes the strain-hardening coefficient; *ε* is defined as the accumulated inelastic strain; *u* is the hardening exponent; *C* corresponds to the strain-rate sensitivity parameter; $\dot{\varepsilon }$ is the equivalent plastic strain rate; ${\dot{\varepsilon }}_{0}$ corresponds to the characteristic strain rate for the material; *T*_0_ is the ambient temperature, typically taken as 20 °C; *T*_melt_ denotes the fusion temperature of the material; and *m* is the parameter that quantifies the rate of heat-induced strength reduction. The constitutive parameters for the titanium alloy are summarized in Table 2.

In this work, two comparative simulation schemes were designed to evaluate ultrasonic vibration-assisted drilling against conventional drilling. For the conventional drilling case, the spindle speed was set to 1200 r/min with a feed of 0.05 mm/r. Building on these conditions, the ultrasonic vibration-assisted drilling simulation incorporated axial vibration parameters, with its vibration frequency set to 20 kHz and amplitude set to 6 μm. Both sets of simulations maintain the same meshing accuracy, and the three directions of X, Y and Z were subjected to fully constrained boundary conditions. The drilling depth is set to 4 mm. The Usui model using metal cutting is the tool wear model.

## 5. Experiment and Identification

### 5.1. Experimental Equipment and Setup

**Figure 10:**
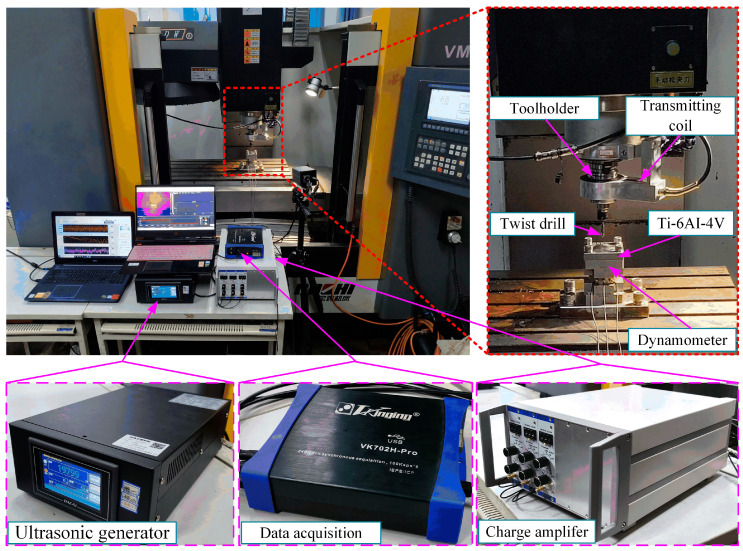
Ultrasonic vibration-assisted drilling experimental equipment.

**Figure 11:**
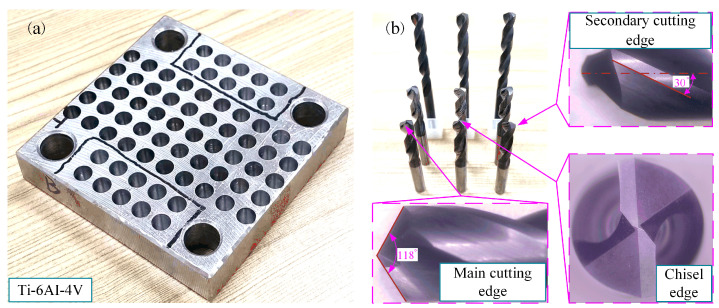
Experimental workpiece and tool: (**a**) Ti-6AI-4V workpiece, (**b**) Φ6 twist drill.

The experimental setup was based on the VMC850B high-precision vertical machining center (Bochi Machine Tool Group Co., Ltd., Baoji, China), as illustrated in Figure 10. This machine tool offers a maximum spindle speed of 8000 r/min and achieves a positioning accuracy of ±0.008 mm. Equipped with an ultrasonic vibration drilling system, consisting of an ultrasonic generator, transmit coil, and ultrasonic tool holder. (High Beam Energy Nanotechnology Co., Ltd., Jinan, China). Cutting force signals were captured in real time using a three-component dynamometer operating at a sampling rate of 10 kHz. A conventional Φ6 mm twist drill was employed for the tests. The workpiece material was Ti-6Al-4V alloy with dimensions of 80 × 80 × 20 mm. Table 3 summarizes the mechanical characteristics of the tool and workpiece materials. Each hole was drilled to a depth of 19 mm, and for every parameter setting, five consecutive holes were produced to ensure repeatability and reliability. The final experimental results were obtained by averaging the measurements from these repetitions. The tool and workpiece used in the experiments are depicted in Figure 11, while Table 4 presents the parameters for finite element simulation and experiment.

### 5.2. Identification of Model Parameters

The experimental results show that the shear stress remains basically stable under different cutting layer thicknesses and cutting radii. Additionally, there is a linear relationship between the friction force on the minor cutting edge and the drilling depth, and the magnitude of the reinforcement coefficient is obtained by fitting the polynomial function.

The unknown parameter shear stress *τ*_s_ in the force model needs to be identified through the constraint relationship between the tool’s cutting angle and the cutting force components. Firstly, the dynamometer is used to obtain the drilling thrust force under different drilling parameters and different drilling methods. In the case where the tool’s geometric parameters and processing parameters are known, the shear stress is regarded as an unknown quantity, and a polynomial function related to the shear stress value can be acquired by substituting the remaining known parameters. The shear stress was inferred from the thrust force data obtained through multiple sets of tests, and the mean value *τ*_s_ = 602.5 MPa was adopted. By comparing the Refs. [33,34], it is shown that the obtained shear stress meets the theoretical and experimental requirements, indicating the reliability of the test results.

**Figure 12:**
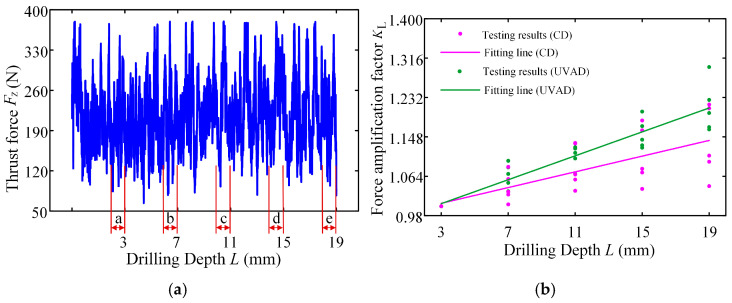
Thrust force curves and fitting function images of different drilling depths: (**a**) thrust force curves of ultrasonic vibration-assisted drilling; (**b**) the variation law of force amplification factor.

Considering that the minor cutting edge has not yet participated in the cutting during the drilling stage, a penetration depth of 2.5 mm corresponds to the point of complete engagement for the main cutting edge, and the obtained thrust force is divided into five zones along the drilling depth, as shown in Figure 12a. Calculate the average axial force in each area separately, where the average force in area a is approximately 193.88 N, and the average force in area e is approximately 237.95 N. Then, divide the average force in each area by the average force in area a to obtain the relative growth rate of axial force as the drilling depth increases.

The thrust forces of different drilling methods obtained by the test are fitted, and the FAFs of the two drilling methods are obtained, respectively. The *K*_L_ fitting curve of the FAF under different drilling methods is shown in Figure 12b; the FAF of ultrasonic vibration-assisted drilling is *K*_L_ = 0.0510, and that of conventional drilling (*A* = 0) is *K*_L_ = 0.0335. The reason for the above results is that the cutting path of the main cutting edge increases during ultrasonic vibration-assisted drilling, and the friction time between the minor cutting edge and the hole wall is prolonged under the condition of reciprocating motion, which leads to the increase rate of its thrust force with depth higher than that of convention drilling, that is, the FAF of ultrasonic vibration-assisted drilling is greater than that of convention drilling.

By verifying the FAF and substituting the established drilling force analytical model, the influence of drilling depth on the drilling force can be quantified. After clarifying the allowable thrust force and allowable torque of the twist drilling tool, the maximum ultimate drilling depth for precision hole machining can be obtained.

## 6. Analysis and Discussion

### 6.1. Drilling Deformation and Force

As a common external chip discharge hole processing method, twist drilling relies on the drill’s flute structure to affect the smoothness of chip evacuation, which in turn plays a critical role in the stability of the whole processing process. The chips generated by the change in drilling time under different drilling methods are shown in Table 5.

During titanium alloy machining, the chip shape changes with machining parameters under conventional drilling conditions. It can be seen that the chips produced during conventional drilling are usually not easy to break and take on a long, strip-like shape. With greater drilling depth, chip length extends progressively and may even form spiral coils. In practical machining, elongated chips tend to wrap around the drill, which not only accelerates tool degradation but can also compromise the quality of the machined hole. As a result, these long helical chips are highly likely to scratch the machined surface during evacuation, thereby reducing dimensional accuracy and impairing the integrity of the hole wall.

The variations in chip morphology under ultrasonic vibration-assisted drilling are as follows: As illustrated in the figure, when stable ultrasonic conditions are maintained, the produced chips are readily fragmented. This occurs because the chip root experiences higher strain and localized stress concentration, which promotes fracture. Even as drilling depth increases, the dynamic action of ultrasonic vibration continues to suppress the generation of long chips, ensuring that they break promptly during machining, and avoiding the accumulation of long spiral chips. At the same time, ultrasonic vibration can also reduce the extrusion action during the chip discharge process, making the chips loose, thus forming loosely folded short bands. These loose chips not only reduce chip entanglement but also improve chip evacuation and enhance the stability of the cutting process.

As evidenced by both numerical and experimental results, the peak chip length generated by ultrasonic vibration-assisted drilling is about 1/2 of that of conventional drilling, and during the test, the chips produced by the whole process of ultrasonic vibration-assisted drilling are mostly chips and flake chips. By minimizing frictional forces between the tool, chips, and workpieces, ultrasonic vibration-assisted drilling can effectively reduce tool wear and reduce surface defects during machining.

**Figure 13:**
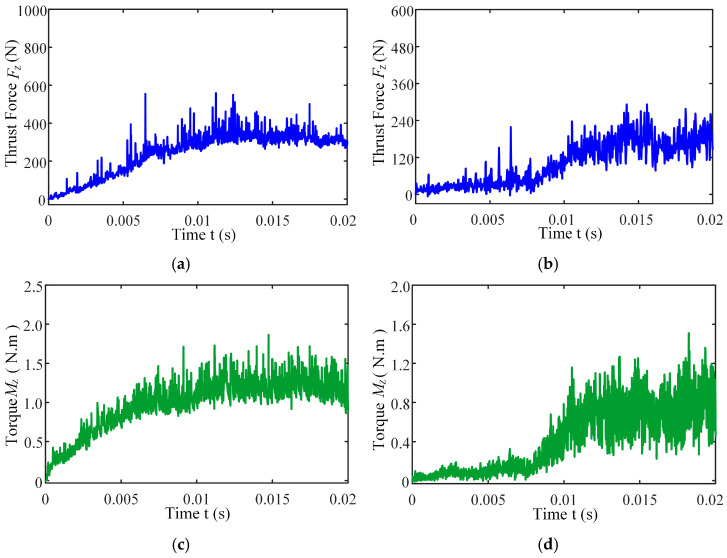
Instantaneous thrust force and torque change curves under different drilling methods: (**a**) thrust force of CD; (**b**) thrust force of UVAD; (**c**) torque of CD; (**d**) torque of UVAD.

Figure 13 presents the instantaneous thrust force and torque data patterns for different drilling techniques over time. Simulation results indicate that conventional drilling produces an average thrust force of approximately 327.3 N and an average torque of 1.20 N·m, whereas ultrasonic vibration-assisted drilling at 20 kHz yields significantly lower averages of about 193.9 N and 0.70 N·m, respectively.

In conventional drilling, the thrust force and torque gradually increase before reaching a steady state with minor fluctuations. In contrast, under ultrasonic vibration-assisted conditions, the force and torque curves exhibit a slight initial rise followed by a stable stage characterized by higher-amplitude oscillations compared with conventional drilling. Because the cutting layer thickness in ultrasonic vibration-assisted drilling varies periodically, while the cutting layer thickness in conventional drilling is a constant value, which is half of the feed amount. This results in a larger oscillation amplitude of the instantaneous force curve in the ultrasonic vibration drilling process compared to conventional drilling. However, due to the influence of ultrasonic vibration, the axial force and torque decreased by 40.8% and 41.7%, respectively.

### 6.2. Model Validation

**Figure 14:**
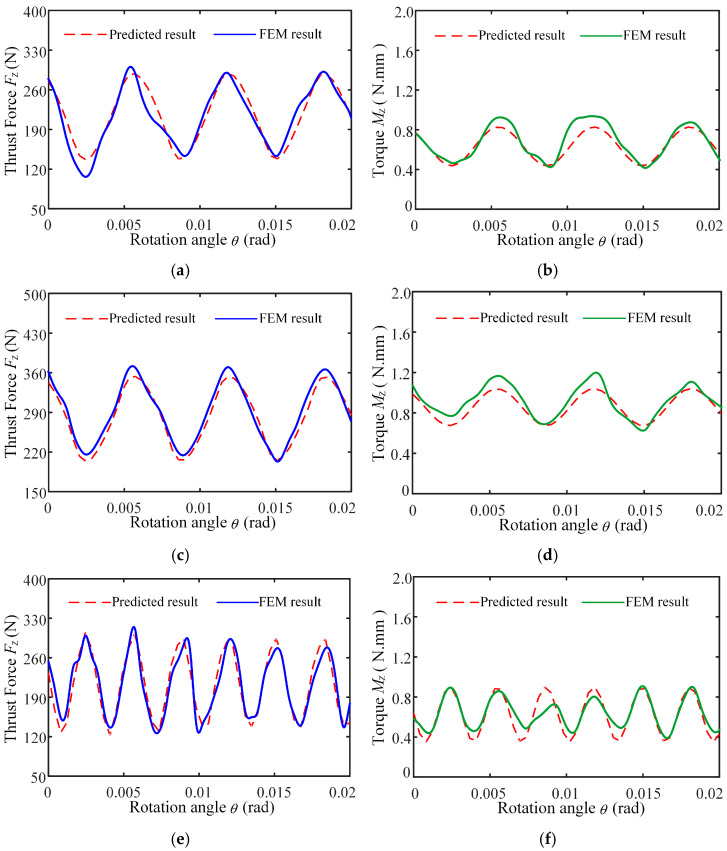
Instantaneous thrust force and torque change curves of analytical model prediction and finite element simulation: (**a**) Instantaneous drilling thrust force of *n* = 1200 r/min, *f*_r_ = 0.05 mm/r, *A* = 6 μm, *f* = 20,010 Hz; (**b**) Instantaneous drilling torque of *n* = 1200 r/min, *f*_r_ = 0.05 mm/r, *A* = 6 μm, *f* = 20,010 Hz; (**c**) Instantaneous drilling thrust force of *n* = 1200 r/min, *f*_r_ = 0.07 mm/r, *A* = 6 μm, *f* = 20,010 Hz; (**d**) Instantaneous drilling torque of *n* = 1200 r/min, *f*_r_ = 0.07 mm/r, *A* = 6 μm, *f* = 20,010 Hz; (**e**) Instantaneous drilling thrust force of *n* = 600 r/min, *f*_r_ = 0.05 mm/r, *A* = 6 μm, *f* = 20,010 Hz; (**f**) Instantaneous drilling torque of *n* = 600 r/min, *f*_r_ = 0.05 mm/r, *A* = 6 μm, *f* = 20,010 Hz.

Using the control variable method, the effect of each parameter on thrust force was systematically investigated. The specific settings for the single-factor experiments are listed in Table 5. To validate the accuracy of the proposed analytical model for drilling force, three sets of simulation results were selected for comparison. The predicted instantaneous thrust force and torque curves obtained from the analytical model and finite element simulations under varying machining parameters are illustrated in Figure 14. The mean absolute errors under varied machining conditions between the model predictions and the simulated thrust force and torque are 7.87% and 6.26%, respectively, and the peak absolute deviation of peaks and troughs are 8.67% and 14.46%, respectively. The results show that the trend and peak of the model’s force predictions demonstrate a strong correlation with the finite element simulation results, and the established model is reliable.

**Figure 15:**
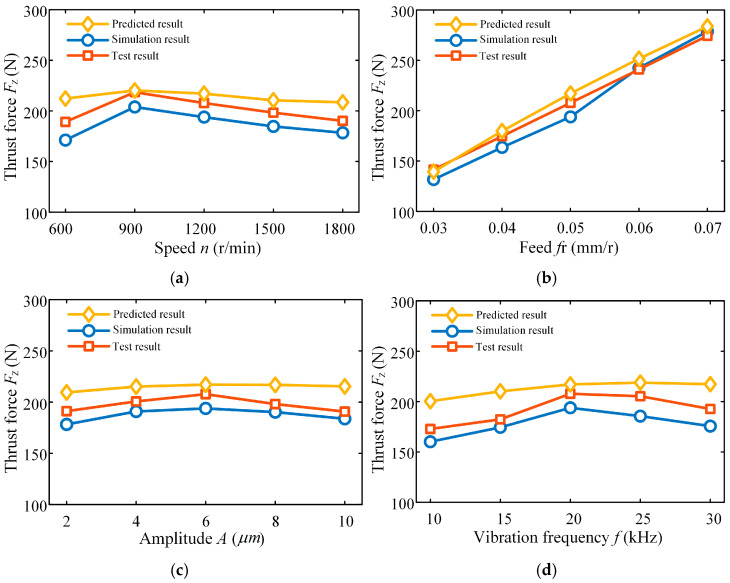
Effects of ultrasonic vibration-assisted drilling variables on force generation: (**a**) thrust force variation law under different spindle speeds; (**b**) thrust force variation law under different feeds; (**c**) Thrust force variation law under different amplitudes; (**d**) thrust force variation law under different frequencies.

Figure 15 illustrates the relationship between drilling force and process parameters in ultrasonic vibration-assisted drilling, demonstrating their significant impact on thrust force. Specifically, Figure 15a shows how spindle speed affects the thrust force. Results show that the thrust force grows within the speed range of 600–900 r/min, but gradually declines as the speed rises from 900 to 1800 r/min. At lower spindle speeds (600–900 r/min), the extended contact time between the main cutting edge and the workpiece, combined with the relatively low vibration frequency per unit time, leads to higher cutting loads and consequently greater thrust force. As the spindle speed continues to rise, high-speed cutting reduces the tool–workpiece contact time while simultaneously decreasing the number of reciprocating cutting actions per unit time.

Figure 15b presents the influence of feed on the thrust force during ultrasonic vibration-assisted drilling. As shown by the data, the thrust force rises markedly with increasing feed, and the growth rate becomes progressively steeper. This behavior can be explained by the fact that a higher feed rate increases the cutting thickness removed by the drill per revolution when machining titanium alloy under ultrasonic assistance, which directly leads to a greater thrust force.

The variation law of thrust force with amplitude of ultrasonic vibration-assisted drilling is shown in Figure 15c. As amplitude increases, the drilling thrust initially rises, followed by a subsequent decline. A larger amplitude corresponds to increased relative displacement between the tool and the workpiece, the tool periodically detaches from the workpiece surface, thereby reducing the average cutting force, and the tool has more time in the non-cutting state during each vibration cycle, so the time for the tool to actually participate in cutting per unit time is reduced, resulting in a decrease in thrust force. At the same time, a larger amplitude reduces the instantaneous cutting thickness, and the cutting force is directly proportional to this thickness; further, a larger amplitude enhances the variation in dynamic cutting thickness, thereby reducing the thrust force.

Amplitude is a unique process parameter of ultrasonic vibration-assisted drilling, and the increase in amplitude causes chips to break easily, making chip formation and discharge smoother, and reducing friction and material deformation resistance, thus reducing the resistance that the tool needs to overcome during the cutting process.

Figure 15d illustrates the influence of vibration frequency on thrust force in ultrasonic vibration-assisted drilling. The results reveal that thrust force initially rises with increasing frequency, reaching a maximum at 25,010 Hz, after which it declines as the frequency continues to increase up to 30,010 Hz. Regarding chip morphology, higher frequencies promote chip fragmentation, producing mainly small debris, which facilitates chip evacuation. Moreover, elevated frequencies enhance the repeated micro-cutting and extrusion on the tool–workpiece interface, thereby reducing surface roughness. Nonetheless, theoretical analysis suggests that excessively high frequencies can accelerate tool wear and elevate drilling temperatures, ultimately impairing tool life and machining stability.

## 7. Conclusions

This study explores the utilization of ultrasonic vibration-assisted drilling in the machining of titanium alloys and establishes a predictive model for drilling force. The model incorporates tool geometry, the cutting radius scale effect, dynamic cutting thickness, and drilling depth, and is formulated through a combination of theoretical derivation, computational modeling and empirical verification. Furthermore, the effects of process parameters on drilling force, chip formation, and machining quality are systematically analyzed. Below is a summary of the primary conclusions:

Through kinematic analysis of ultrasonic vibration-assisted drilling, a comprehensive drilling force model was constructed that accounts for tool geometry, scale effects of the cutting radius, dynamic cutting thickness, and drilling depth.

Across varying machining conditions, the mean absolute deviations between model predictions and simulated thrust force and torque were 7.87% and 6.26%, respectively, while the maximum absolute errors at peak and trough values reached 8.67% and 14.46%. These results confirm that the proposed model reliably predicts instantaneous thrust force and torque in ultrasonic vibration-assisted drilling.

The combined effect of a vibration frequency of 30 kHz and an amplitude of 10 μm significantly improves chip morphology by promoting fragmentation and minimizing entanglement. With increasing drilling depth, both thrust force and torque show a concurrent increase, with the force reinforcement coefficient of ultrasonic drilling determined as *K*_L_ = 0.0510.

In comparison to the conventional approach, ultrasonic vibration-assisted drilling effectively lowers thrust force and torque, with reductions of approximately 40.8% and 41.7%, respectively. The drilling force decreases initially with higher spindle speed, increases with greater feed, and exhibits a rise–fall trend with variations in vibration frequency and amplitude.

This article establishes an analytical model for drilling force considering tool geometric parameters, cutting radius scale effects, dynamic cutting thickness, and drilling depth. However, the unknown parameter of shear stress *τ*_s_ in the force model needs to be identified through the constraint relationship between the tool’s cutting angle and cutting force components. Therefore, experiments are required to identify the shear stress *τ*_s_ of different materials.

## Figures and Tables

**Table 1:** Geometric parameters of the tool.

Diameter (mm)	Chisel Edge Width (mm)	Margin Width (mm)	Land Height (mm)	Point Angle (°)	Helix Angle (°)
6	1	0.1	0.1	118	30

**Table 2:** Constitutive model coefficients of Ti-6Al-4V materials [32].

*A* (MPa)	*B* (MPa)	*C*	*m*	*u*	*T*_melt_ (°C)	*T*_0_ (°C)	${\dot{\varepsilon }}_{0}$
783	498	0.028	1.0	0.28	1578	20	0.01

**Table 3:** Material properties of WC and Ti-6Al-4V.

Material	Density *ρ* (g/cm^3^)	Elastic Modulus E (Gpa)	Poisson Ratio μ
WC	14.5	600	0.31
Ti-6Al-4V	4.5	113.8	0.342

**Table 4:** FEM simulation-experiment parameters.

Parameter	Parameter Levels					Reference Value
Speed *n* (r/min)	600	900	1200	1500	1800	1200
Feed *f*_r_ (mm/r)	0.03	0.04	0.05	0.06	0.07	0.05
Amplitude *A* (μm)	2	4	6	8	10	6
Vibration frequency *f* (Hz)	10,010	15,010	20,010	25,010	30,010	20,010

**Table 5:** Comparison of chip morphology between UVAD and CD.

	Step 5000	Step 10,000	Step 15,000	Step 20,000
Chip morphology (CD)	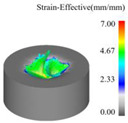 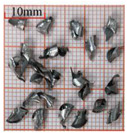	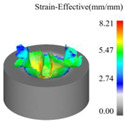 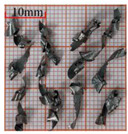	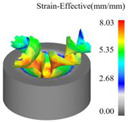 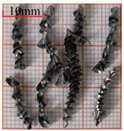	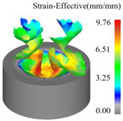 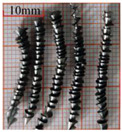
Chip morphology (UVAD)	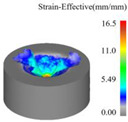 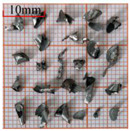	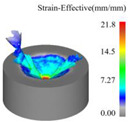 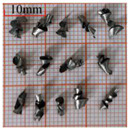	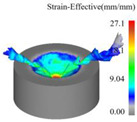 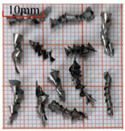	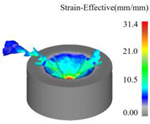 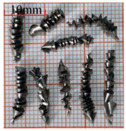

## Data Availability

The original contributions presented in this study are included in the article. Further inquiries can be directed to the corresponding author.
